# Generating large-scale real-world vehicle routing dataset with novel spatial data extraction tool

**DOI:** 10.1371/journal.pone.0304422

**Published:** 2024-06-21

**Authors:** Hina Ali, Khalid Saleem

**Affiliations:** 1 Department of Computer Sciences, Quaid-i-Azam University, Islamabad, Pakistan; 2 Department of Computer Sciences, National University of Modern Languages Islamabad, Islamabad, Pakistan; Southwest Jiaotong University, CHINA

## Abstract

This study delves into the critical need for generating real-world compatible data to support the application of deep reinforcement learning (DRL) in vehicle routing. Despite the advancements in DRL algorithms, their practical implementation in vehicle routing is hindered by the scarcity of appropriate real-world datasets. Existing methodologies often rely on simplistic distance metrics, failing to accurately capture the complexities inherent in real-world routing scenarios. To address this challenge, we present a novel approach for generating real-world compatible data tailored explicitly for DRL-based vehicle routing models. Our methodology centers on the development of a spatial data extraction and curation tool adept at extracting geocoded locations from diverse urban environments, encompassing both planned and unplanned areas. Leveraging advanced techniques, the tool refines location data, accounting for unique characteristics of urban environments. Furthermore, it integrates specialized distance metrics and location demands to construct vehicle routing graphs that represent real-world conditions. Through comprehensive experimentation on varied real-world testbeds, our approach showcases its efficacy in producing datasets closely aligned with the requirements of DRL-based vehicle routing models. It’s worth mentioning that this dataset is structured as a graph containing location, distance, and demand information, with each graph stored independently to facilitate efficient access and manipulation. The findings underscore the adaptability and reliability of our methodology in tackling the intricacies of real-world routing challenges. This research marks a significant stride towards enabling the practical application of DRL techniques in addressing real-world vehicle routing problems.

## Introduction

Vehicle route optimization is pivotal in modern logistics, aiming to enhance operational efficiency and cost-effectiveness. Efficient routing ensures timely delivery and reduces resource consumption, aligning with sustainability goals. Vehicle Routing Problem (VRP) being incepted by George Dantzig and John Ramsar in 1959 [1] and grounded in the realms of combinatorial optimization and integer programming, aims to address questions such as, ‘How can we determine the most efficient routes for a fleet of vehicles to deliver goods to a specified group of customers?’ [2]. However, the modern landscape of supply chains, marked by fluctuating demand and diverse vehicle capacities, presents significant challenges to the traditional routing methods commonly employed in the Operations Research (OR) community. Additionally, in OR, regardless of whether the VRP instance consists of 20 or 1000 nodes, only a few instances are typically required in experimentation. This renders the conventional OR dataset unsuitable for DRL based setup. The Vehicle Routing Problem (VRP) encompasses a fleet of vehicles, various locations to visit, and customer demands. These locations are categorized into depots and customers, with depots serving as the starting points for vehicle routes. Each location, excluding the depot, signifies a demand that must be met. Key components of a VRP dataset include the number of vehicles (capacitated or incapacitated), customer locations, customer demands, and distances between locations. Dataset generation typically follows one of three approaches for constructing the transportation network: (i) generating synthetic data-based transportation networks, as seen in studies such as [3–5]; (ii) simulating transportation networks, as evidenced by research like [6–8]; or (iii) constructing transportation networks using real-world data, as demonstrated by works such as [9] for test datasets, along with [10, 15] The most prevalent approach for crafting a transportation network involves the use of synthetic data. In this scenario, data is dynamically generated within a unit square with coordinates ranging from [0, 1] [0, 1]. Euclidean distance is utilized to measure cost of path between randomly chosen coordinates within this square. In a simulation setting, a simulator is employed to emulate real-world traffic conditions. The simulator generates data instances based on the guidelines established through the synthetic data generation process. It continuously monitors vehicle movement to meet demand by considering the vehicle’s dynamic position and locations.

In research, real-world datasets commonly incorporate geospatial coordinates and path costs, often based on Euclidean distances or those provided by mapping service APIs. However, these datasets present several limitations [6, 15]. Firstly, due to their limited size, these datasets are primarily suited for OR studies or as test/validation sets for deep learning models (limitation 1). Secondly, all delivery points are treated equally without consideration for factors such as order types, frequency, or the nature of locations (e.g., residential versus commercial), significantly impacting the optimization of last-mile delivery routes (limitation 2). Finally, the provided locations may not align with actual street addresses; rather, they are often randomly generated points sourced from traffic datasets, possibly representing non-existent locales, (limitation 3).

Currently, there is a notable absence of real-world Capacitated VRP(CVRP) datasets to address these limitations. To bridge this gap, it is crucial to develop a robust tool for extracting and curating real-world data, particularly tailored for data-intensive VRP frameworks. Our proposed solution is the Spatial Data Extraction and Curation Tool (SDECT), which harnesses mapping service APIs to extract VRP datasets from any global region. SDECT effectively tackles the limitations inherent in current VRP datasets for data-intensive applications by presenting data in a graph format. Moreover, the generated dataset is deemed suitable for a wide array of studies, accommodating both graph-based and non-graph-based analyses.

**Fig 1:**
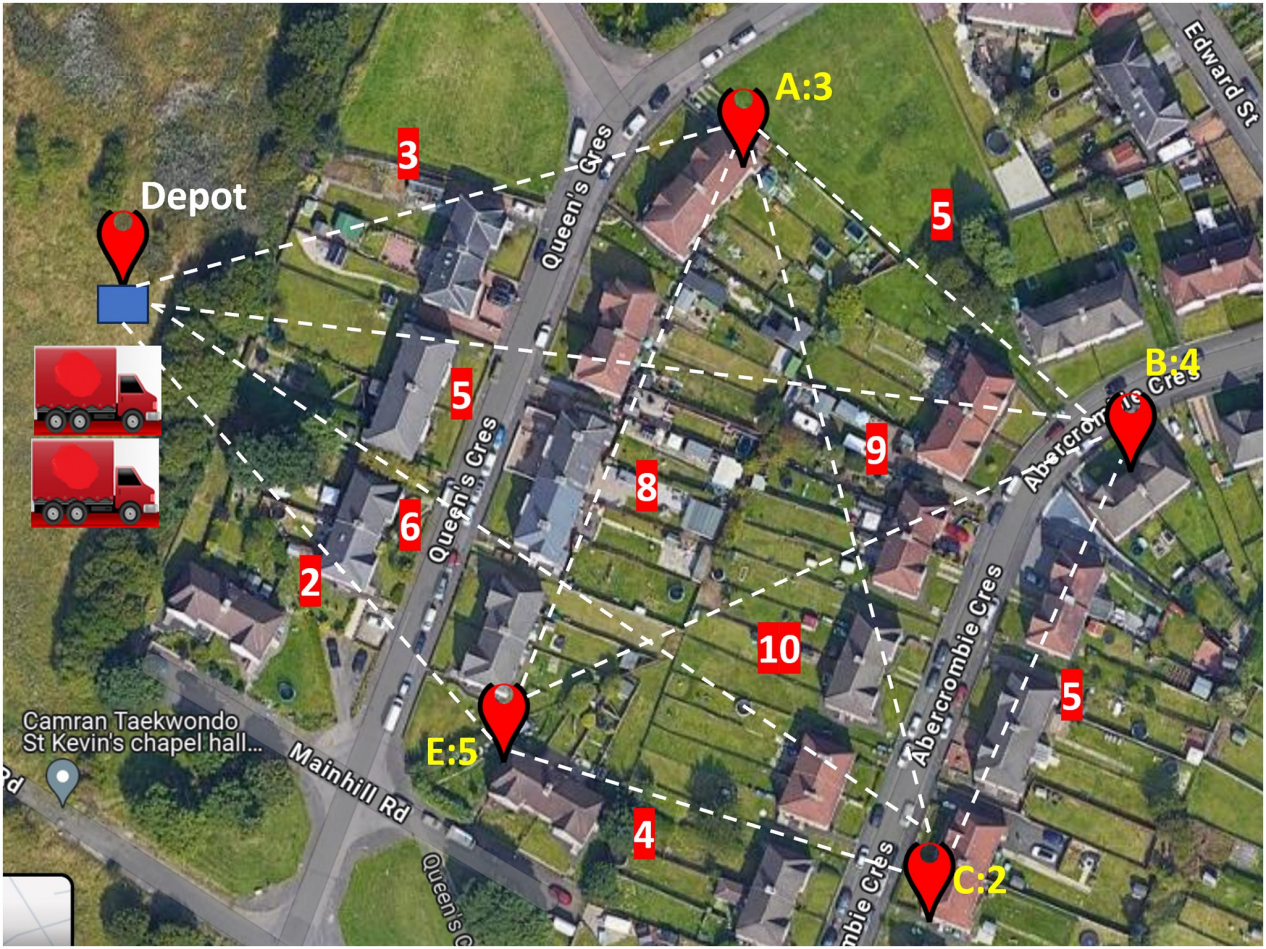
Illustration of a graph instance with 5 location nodes, depicting edge weights as distances. Depot marked in blue, delivery locations marked in red.

**Fig 2:**
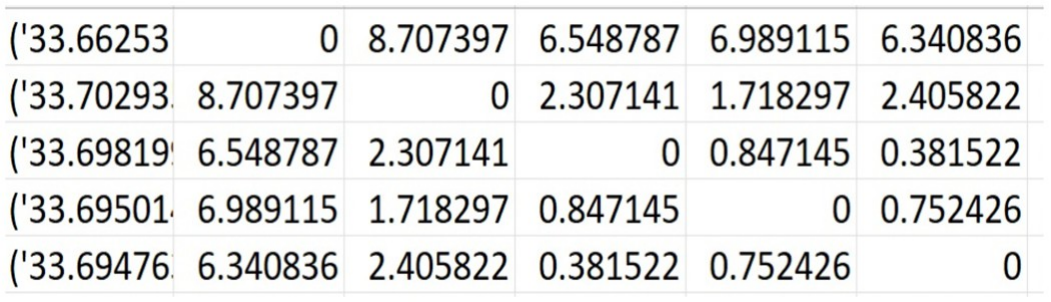
Visualization of a distance matrix for a 5-node graph. The first row corresponds to depots, while the subsequent rows represent delivery locations.

To facilitate a deeper understanding of the dataset, we present a glimpse into its structure through two illustrative figures. In Fig 1, a concise representation of a graph instance is provided, offering insight into the dataset’s composition. The graph comprises five nodes, each serving a unique purpose. The central node, denoted as the depot, is distinguished by a blue square, serving as the hub for vehicle dispatch. Surrounding it are delivery/customer locations, identified by red markers labeled in the format [alphabet:number]. Here, alphabets A, B, C, and E denote the respective delivery/customer locations, with accompanying numerical values representing the associated demands to be fulfilled by the fleet of vehicles stationed at the depot. In Fig 2, we present the distance matrix of a graph instance consisting of five nodes. The first column details the latitude and longitude coordinates of all locations, while the subsequent columns specify the distances between these locations in their respective cells. The initial row is dedicated to depicting the latitude and longitude of the depot, along with its distances from delivery locations. Following rows continue this pattern, showcasing the coordinates and distances from each node to every other node.

Notably, each location is interconnected, forming a network of links or edges. The numerical values adjacent to these links signify the distance, measured in kilometers, between the connected locations. Scalability of SDECT is worth mentioning, which seamlessly adapts to graphs of varying sizes.

SDECT showcases exceptional versatility beyond its application in vehicle routing scenarios. It extends its capabilities to tackle a spectrum of optimization challenges across various domains. For example, it excels in strategically placing cell towers, optimizing routes for fiber optic cables, coordinating drone-based internet delivery, and establishing emergency communication networks. Telecommunications companies can harness the power of SDECT to enhance their operations significantly. Moreover, by optimizing routes for repair crews, SDECT contributes to increased efficiency in maintenance operations, thereby benefiting the telecommunications sector as a whole. This tool empowers researchers to validate their models in real-world scenarios, thereby enhancing the practical applicability of their findings.

Below, we outline the key contributions of our work.

1. Our foremost contribution revolves around the creation of SDECT, a sophisticated tool, designed to extract and organize real-world datasets using mapping service APIs. This comprehensive dataset not only includes locations of depots and customer’s delivery points, but also provides an adjacency matrix detailing distances between these locations. To further enhance its utility, we have categorized these locations into eight primary types, encompassing offices, banks, commercial hubs, residential neighborhoods, hospitals, places of worship, educational institutions, and miscellaneous areas. Moreover, each delivery point is mapped with its unique set of demands, ensuring a granular understanding of the delivery landscape and address limitations 1 and 2.
2. To make dataset of locations precise, we utlized shape files from mapping service providers. Only shapes files of building and landcover are selected to ensure that extracted locations corresponds to actual street addresses to address limitation 3
3. Finally, it can be used in both planned and unplanned areas to create location graphs of required sizes. Consequently, it emerges as a crucial instrument for generating dataset that facilitate in optimizing transportation logistics in any real-world setting.

The organization of this paper is as follows. In beginning we offer a concise overview of the existing literature in the field, highlighting relevant methodologies. Its is followed by methodology, that delineates the tool’s mechanisms for extracting, organizing, and refining spatial data, elucidating its pivotal role in the domain. The result and discussion part of study outlines the experimentation and the data utilized along with the approach taken to achieve results. Finally, the concluding section captures the main findings resulting from the experiments carried out and subsequent discussions.

## Current state of work

The VRP has been a subject of interest in OR for many years. Since the VRP is a specialized version of the Traveling Salesman Problem(TSP), the basic dataset characteristics and generation method are comparable. The ongoing research effort is reflected in the OR dataset repository CVRPLIB [11], which includes datasets spanning from 1987 to the present. Two of these benchmark datasets are listed in the OR section of Table 1. The reason for including OR datasets is that they represent the fundamental criteria for creating additional VRP datasets.

**Table 1:** Table list of benchmark data-sets of operation research and deep learning.

Size of Problem (N)	Type of Dataset	Limitations	Reference
**Operation Research(OR) (CVRPLIB)**			
N = 100	Synthetic Solomon Instance	• Very Small in size • Can be used as test instances in DL only • No real-time distance and traffic information	[19]
N = 200, 400, 600, 800, 1000	Synthetic Instance	• Same as above	[20]
N = 25, 500, 100	Synthetic Instance	• Same as above	[21]
N = 25, 500, 100	Real-World	• Same as above	[22]
**Deep Learning (DL)**			
N = 10, 20, 50, 100	Synthetic Instances	• Euclidean distance • No real-time distance and traffic information	[12]
N = 10, 20, 50, 100	OR, Synthetic and Real-time data	• Average traffic speed taken • Location Chosen randomly • No information about categorization of location basis of types	[14]
N = 10 to 100	Synthetic and Real-time data	Same as [14]	[15]
N = 10, 20, 50, 100	Synthetic and Real-time data	• Same as [12]	[4]
N = 20, 50, 100	Synthetic collected through Simulator	• Same as [4, 12]	[23]
N = 20, 50, 100	Synthetic Data-set	• Same as [4, 12]	[24]
N = 20, 50, 100	Synthetic Data-set	• Same as [4, 12]	[25]

The second section of Table 1 contains VRP datasets used in deep learning setups and mentioned as benchmarks in [4, 12]. the initial attempt to create a VRP dataset for deep learning occurred in 2018 [12].

A review of the articles listed in Table 1 reveals that most datasets are synthetically generated, eliminating the need for a data extraction tool. Geo spatial dataset based research studies are categorized into two groups: 1) OR and 2) deep learning. Datasets utilized for solving routing problem in OR are often constrained in size, rendering them inadequate for deep learning applications. However, they can serve as valuable resources for testing in deep learning, facilitating comparative analyses. In one such OR based real-world VRP study, researchers generated a transportation network for transit services using 25,843 trip requests. The dataset includes the location coordinates of pickup and drop-off locations, along with pickup times. Researchers utilized OSM for road network data and travel time calculations, employing the shortest travel time to construct the road network. [13] In the context of deep learning, real-world datasets are mentioned in [14]. They have generated routes from traffic data, which includes road network data but lacks information about street data, location types, and other relevant details. same datset is used in another approach thus having Same limitations [15] In another example, a simulated dataset for the electric vehicle routing problem was created in the form of a transportation network with 30 customers and five vehicles, where all parameters except vehicle dimensions were simulated [2]. This information about real-world dataset might exist within specific companies that have already adopted these tools to assist in planning tours. However, obtaining generalize data for scientific use, especially in vehicle routing domain is challenging, As companies are hesitant to fully disclose their operations [16]. Therefore most of research in this domain is carried out on simulated data [17]. Unavailability of real world data is evident in field of automated guided vehicle, wherein utilization of spatial data is adopted in testing phase [18]. The research domain encounters ongoing challenges in overcoming constraints related to obtaining genuine operational data. This underscores the importance of employing innovative approaches to tackle these obstacles and enhance comprehension in the realm of vehicle routing problems and their applications. SDECT has been developed to specifically address the challenge of generating real-world graph datasets tofill the void in dataset generation and curation.

## Methods

Our research is dedicated to extracting vector data in the form of location graphs paired with distance matrices. Initially, we obtain location data with the support of mapping service providers, gathering latitude and longitude coordinates. From these coordinates, we construct graphs by specifying the number of location nodes and generating graphs of particular sizes.

**Fig 3:**
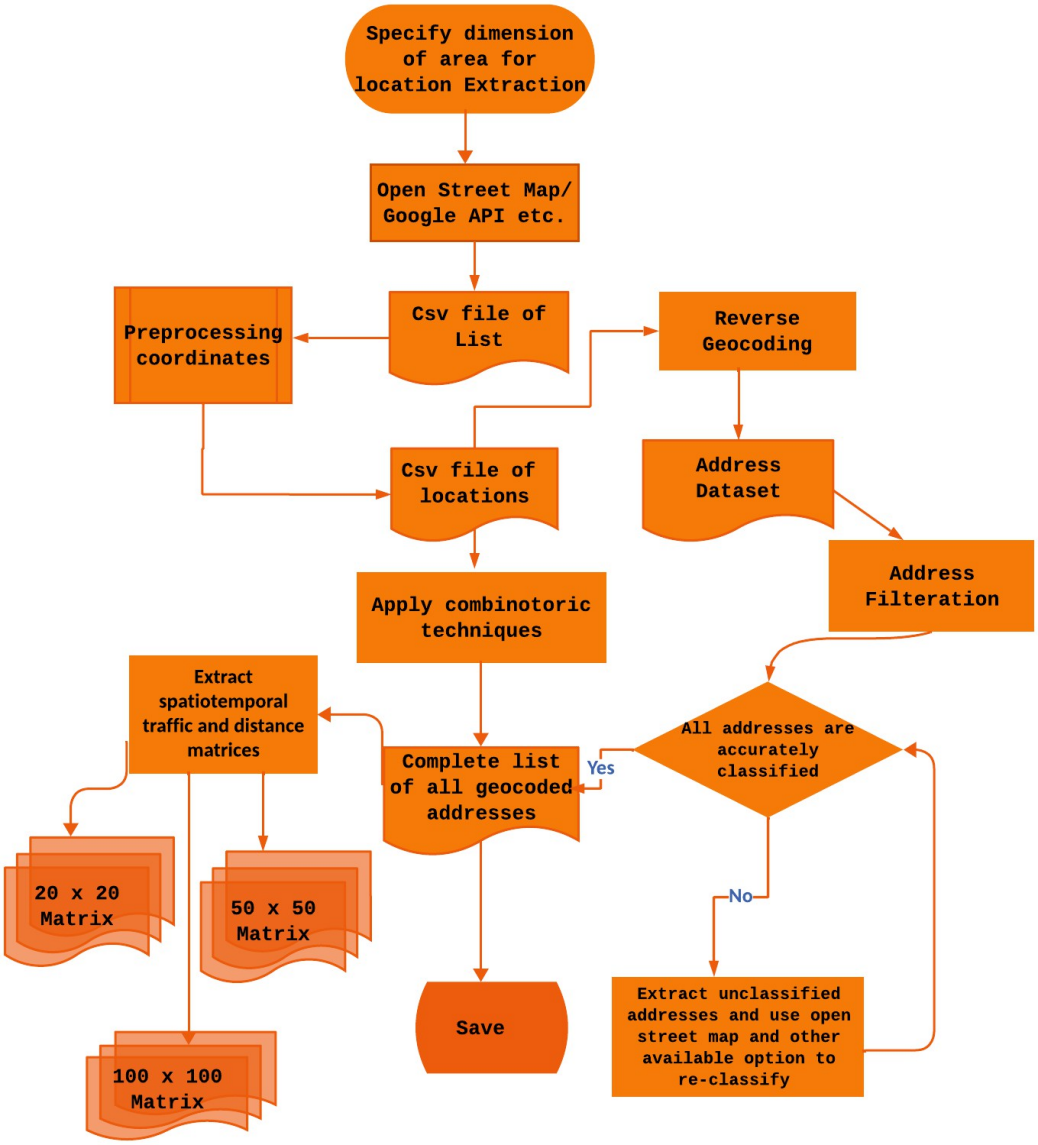
SDECT. A flowchart illustrating the progression of steps within SDECT.

The working of SDECT for dataset extraction and curation is depicted in Fig 3.

Using mapping service direction APIs, we generate adjacency matrices representing the distances between locations in the graph, as showcased in Figs 1 and 2. Before converting the data into location graphs, it undergoes rigorous curation. Initially, we filter out only valid addresses by leveraging shape files of buildings and land cover from OpenStreetMap APIs. This step eliminates unwanted addresses that may be randomly generated within a range of minimum and maximum coordinates, such as those associated with roads, water bodies, and public parks.

Next, the coordinates undergo reverse geocoding using multiple mapping service provider APIs like Google, OSM, and GraphHopper to obtain corresponding addresses. Coordinates that do not yield addresses are discarded. Subsequently, the addresses are subjected to filtration through an address parser to categorize them into eight distinct categories. This categorization process involves filtering addresses based on keywords such as road names, shops, schools, etc. If an address contains a specific keyword, it is allocated to the corresponding category. Additionally, redundant addresses referring to the same location are also discarded to enhance the dataset quality.

SDECT, our proposed method, finds applications in various settings, including urban (both planned and unplanned) and rural areas. In the case of planned urban areas, we divide the city into a grid of square cells, each measuring 2 km * 2 km. These cells, known as sectors, are identified by a combination of a letter and a number (e.g., F-10 and I-10). We select the latitude and longitude coordinates of the northwestern and southeastern corners of these sectors. To illustrate coordinate extraction from unplanned areas, we choose a city characterized by non-uniform territorial division. Despite having administrative divisions, its geographical distribution lacks uniformity, featuring varying sector sizes and irregular shapes. To standardize data extraction, we partition these sectors into smaller rectangles or squares. Then, we extract the latitude and longitude coordinates of the northwestern and southeastern corners of each subdivision for utilization in SDECT.

### Working of spatial data extraction and curation tool

To ensure that the data extracted from unplanned city layout remain consistent with planned city layout, we further divided these layouts in basic 2 * 2 km square kilometer for data extraction. We have chosen this size because (i) riders typically travel 2-3 kilometers, according to the industry standard for ride-hailing services; and (ii) every unit in the selected planned city is 2 kilometers in size. But it is worth noting that size is a customized parameter in this study and it can be altered to meet specific requirement, such as a smaller or larger region of interest.

SDECT has two modules: (i) a location extraction module, and (ii) a route matrix generation module.

#### Location extractions

The essential prerequisites for this module are given below detailed in Tables 2 and 3:

- Determining the country for generating location-based data.
- Basic knowledge of the geographical distribution and division of the selected country.
- Access to a map service provider and direction API key.

After confirming the prerequisites, the process begins with selecting the region of interest, which could encompass planned or unplanned areas within a city. For planned regions, we identify the fundamental division components. Sebsequently, the system require the number of locations coordinates to extract, offering users a wide range of flexibility—from as few as 2 locations to over 10,000,000 locations and beyond. To ensure an even distribution of these locations across the entire city, an equal number of coordinates must be extracted from each basic division. This is achieved using Eq 1, where the total number of locations (n) is divided by the number of basic geographical divisions (m).


$$\text{Number}\;\text{of}\;\text{Locations}=\frac{n}{m}$$ (1)

With the desired number of locations per basic geographical unit established, the location extraction module is activated, utilizing map service provider APIs to extract minimum and maximum coordinates defining specified boundaries. In our case, each “sector” is further divided into 2km × 2km partitions. If the selected region’s area is less than 1 km², it is merged with the nearest region.

This process iterates across all geographical divisions until a predetermined number of locations are extracted from each. Locations lacking an address are promptly discarded to maintain data integrity. Moreover, coordinates repeatedly referring to general addresses, such as city names, are excluded to ensure relevance. The extracted location coordinates undergo address parsing through a dedicated module, leveraging Google Maps and OpenStreetMap APIs for reverse geocoding.

The addresses are then categorized into eight distinct categories, including residential, commercial, official, school, banks, hospitals, and public places. Public places are intentionally omitted from the selected locations as they do not serve as valid delivery points. The categorization process involves keyword searches within the filtered address list to assign locations to their respective categories. In cases where coordinates remain uncategorized, an alternative geocoding service is employed to obtain complete addresses. These addresses undergo further screening and manual correction efforts if necessary, prioritizing data completeness and accuracy.

Tables 2 and 3 display samples of regions and their respective parameters used for extracting coordinates from both planned and unplanned regions. These tables provide specific examples for clarity. The algorithm 1 illustrates the functioning of the location extraction module.

#### Computational complexity

**Proposition 1** *There are three procedures in Algorithm 1. (i) The computational complexity of the procedure “GenerateCoordinates()” of the proposed Algorithm 1 is O(n), (ii) GenerateAddress() is O(n), and (iii) procedure ValidAddress() has constant time complexity*.

**Algorithm 1** An algorithm For Extraction and Filtration of Geo locations

1: **procedure GenerateCoordinates**

2:  **Require:** Area: Predefined, *Limit* ≥ min(GraphSize)

3:  minLat ← min(latitude)

4:  minLong ← min(latitude)

5:  maxLat ← max(longitude)

6:  maxLong ← max(longitude)

7:  **while N ≠Limit do**

8:   Latt ← RandomDistribution (minLat,maxLat)

9:   Long ← RandomDistribution (minLong,maxLong)

10:   Coordinates ← (str(lat) + “,” + str(long))

11:   file ← Coordinates

12: **procedure GenerateAddress**

13:  **Require:** List of coordinates for which addresses are generated.

14:  **if** List of Coordinates **then**

15:   **while** Coordinate in List **do**

16:    Location ← Coordinate

17:    Address ← Geolocator.Reverse(Location)

18:  **else** File Not Found

19:  **end if**

20: **procedure ValidAddress**

21:  **Require:** List Addresses.

22:  **if** List of Addresses **then**

23:   regex ← all addresses with only city name mentioned in it

24:   **if** regex ≠ address **then**

25:    **if** find(’Keyword’) in address **then**

26:     **return** False

27:    **end if**

28:    **return** True

29:   **end if**

30:  **end if**

**Proof 1** *The proof of proposition 1 is given below*.

1. *In the procedure GenerateCoordinates(), the computation of minimum and maximum latitude and longitude coordinates takes constant time, and the process of generating coordinates from these minimum and maximum latitude and longitude coordinates takes O(n) time. Therefore, the overall complexity of this procedure is O(1) + O(n) = O(n)*.
2. *While in procedure GenerateAddress(), calling reverse geocoding on the list of addresses takes O(n) time. Therefore, the overall time complexity of this procedure is O(n)*.
3. *Finally, all operations in the validAddress() procedure are constant time operations, as it filters addresses on the basis of specific keywords. Therefore, it has a time complexity of O(1)*.

***Note that from the above proofs, it can be inferred that the time complexity of Algorithm 1 is O(n)***.

#### Route matrix generation

The proposed algorithm requires the following parameters:

- The size of the graph of delivery points is specified.
- A mapping service provider to extract the distance matrix of specified graph of delivery points.
- A list of filtered location coordinates, where all coordinates must belong to actual street addresses of an entity and be at least 1 km apart from each other.

The constraints associated with this module are:

- Only the coordinates of actual street addresses of entities are used
- All other coordinates belonging to places like parks, roads, and public areas are excluded.
- Locations must be at least 1 km apart from each other.

#### Working

To efficiently load and retrieve coordinate data in memory, the list is divided into equal-sized chunks. These chunks are then combined to form the complete list, which is used to create the distance matrix.

After all addresses are saved in the required file format, the filtered list of coordinates is processed again using algorithm 2 to create distance matrices of specified sizes between source and destination locations. The distance matrix contains the distance in kilometers. algorithm 2 can be used to generate a distance matrix of any size, but for experimentation purposes, we generated distance matrices of sizes 20, 50, 100, 150, 200, 250, 300, and 350 kilometers.

According to research conducted in the industrial setting of a last-mile delivery service provider, the frequency with which orders are repeated is calculated, and these frequencies are used to determine the repetition percentage of orders in the final list. Multiple lists are created i.e. one list for each area, and all location coordinates are saved in these lists.

**Proposition 2** *The computational complexity of algorithm 2 is O*(*n*^2^).

**Proof 2** *The procedure CreateDistanceMatrix() generates graphs of n x n combination of source and destination locations with a complexity of O*(*n*^2^). *The size of graph is specified. The next phase of the procedure requires O*(*n*^2^) *API calls to calculate the distance between sources and destinations. Therefore, the overall complexity of this procedure is O*(*n*^2^).

**Algorithm 2** An Algorithm For Creating Geo-Saptial Distance Graph

1: **procedure CreateDistanceMatrix**

2:  **Require:** GraphSize:VRPsize

3:  Records ← read(DirToRead)

4:  Start = 0, End = *GraphSize*, *N*_*Graph*_ = 1

5:  **while** *N*_*Graph*_ ≤ sizeof(Records)/ GraphSize **do**

6:   locationGraph = location Coordinates of depot

7:   customerLocation = Records[Start:End]

8:   **for** loc in customerLocation **do**

9:    locationGraph.append((loc[latitude], loc[longitude]))

10:   file = open(Directory, w)

11:   writer = writer(file)

12:   Writer ← locationGraph

13:   count = 0

14:   **for** locSource in customerLocation **do**

15:    **for** locDestination in customerLocation **do**

16:     dist = geodesic(loc1, loc2)

17:     locSource ← append(dist)

18:    writer ← locSource

19:   Records ← read(DirToRead)

20:   start + = GraphSize

21:   end += GraphSize

22:   *N*_*Graph*_ += 1

## Results and discussion

Experiments are carried out on workstation equipped with Nvidia GeForce RTX 3060. Two types of datasets i.e. dataset I and dataset II are generated. Dataset I consists of list of coordinates that are extracted and from OSM [26] and Google Map [27]. Location extraction module is utilized for generation and processing of dataset I. Parameter used for extracting locations from both planned and unplanned areas are listed in Tables 2 and 3 respectively. The results of location extraction modules for planned and unplanned areas are listed in Tables 4 and 5 respectively. These tables also affirm generalizability of SDECT which give consistent results across planned and unplanned urban region of a country. Additionally, Table 6 presents the results of the route matrix generation module of the SDECT tool for an urban region in the country.

**Table 2:** Experimental parameters for planned area fix-size.

Experiment	Area	Limit	Range of coordinates(min to max)
Case-1	G-10	1500	33.667126, 73.013042 33.689489, 73.018123
Case-2	G-11	1500	33.657809, 72.995419 33.680881, 73.001942
Case-3	F-10	1500	33.681637, 73.002202 33.703118, 73.008090
…	…	…	………………………
Case-n	F-11	1500	33.672170, 72.984135 33.694554, 72.991740

**Table 3:** Experimental parameters for unplanned area.

Experiment	Area	Limit	Range of coordinates(min to max)
Case-1	Gulzar-e-Hijri	1500	24.937169, 67.140774 24.976558, 67.121766
Case-2	Faisal Cantonment	1500	24.887355, 67.146080 24.926599, 67.162313
Case-3	Feroz Abad Town	1500	24.869617, 67.035313 25.104026, 67.026997
…	…….	…	…………………….
Case-n	Jamshed Town	1500	24.833337, 67.048609 24.887367, 67.052685

**Table 4:** Results of experiments on planned area fix-size.

Experiments	Coordinates		Addresses		Filtered Addresses	
	Number	Time(sec)	Number	Time(sec)	Number	Time(sec)
Case-1	1500	0.041	452	420	418	0.06
Case-2	1500	0.039	1500	783	1493	0.029
Case-3	1500	0.042	1500	755	1421	0.018
Case-n	1500	0.038	1500	751	1464	0.026

**Table 5:** Results of experiments on unplanned area.

Experiments	Coordinates		Addresses		Filtered Addresses	
	Number	Time(sec)	Number	Time(sec)	Number	Time(sec)
Case-1	1500	0.036	1500	723	1432	0.06
Case-2	1500	0.039	1500	749	1461	0.03
Case-3	1500	0.039	1500	801	1497	0.02
Case-n	1500	0.038	1500	739	1454	0.026

**Table 6:** Results of experiments of graph and distance matrix generation.

No.	Graph size	Time(sec)	Total Records	File size (csv)
**1**	**VRP-20**	**0.07**	**100000**	**9 KB**
**2**	**VRP-50**	**0.500**	**10000**	**49 KB**
**3**	**VRP-100**	**1.851**	**100000**	**187 KB**
**4**	**VRP-150**	**4.18**	**100000**	**415 KB**
**5**	**VRP-200**	**7.410**	**100000**	**732 KB**
**6**	**VRP-250**	**11.703**	**100000**	**1.11 MB**
**7**	**VRP-300**	**16.66**	**100000**	**1.59 MB**
**8**	**VRP-350**	**22.97**	**100000**	**2.16 MB**

### Experimentation procedure for planned areas

For planned area, a city with grid iron layout and uniform geographical distribution is selected, which is already divided into zones and sectors, and further into sub sectors. Each division in selected city is of square of size 2km * 2km.

To exhibit the Location Extraction Module’s efficiency, four scenarios representing distinct sectors within the chosen city are provided as examples. Table 2 details the experimental parameters, encompassing experiment numbers, area names, extracted coordinate quantities per area, and their respective coordinate ranges. The outcomes for the planned area are presented in Table 4. Each column is bifurcated: one part displays the total location count, while the other illustrates the time taken by the location extraction module to generate these figures. In Table 4, the initial column showcases the generated coordinates, followed by address generation and subsequent address filtration. Notably, Case-1 yields a lower address count compared to other cases in Table 4 due to a significant portion of the area being occupied by a park, resulting in fewer available delivery addresses.

**Fig 4:**
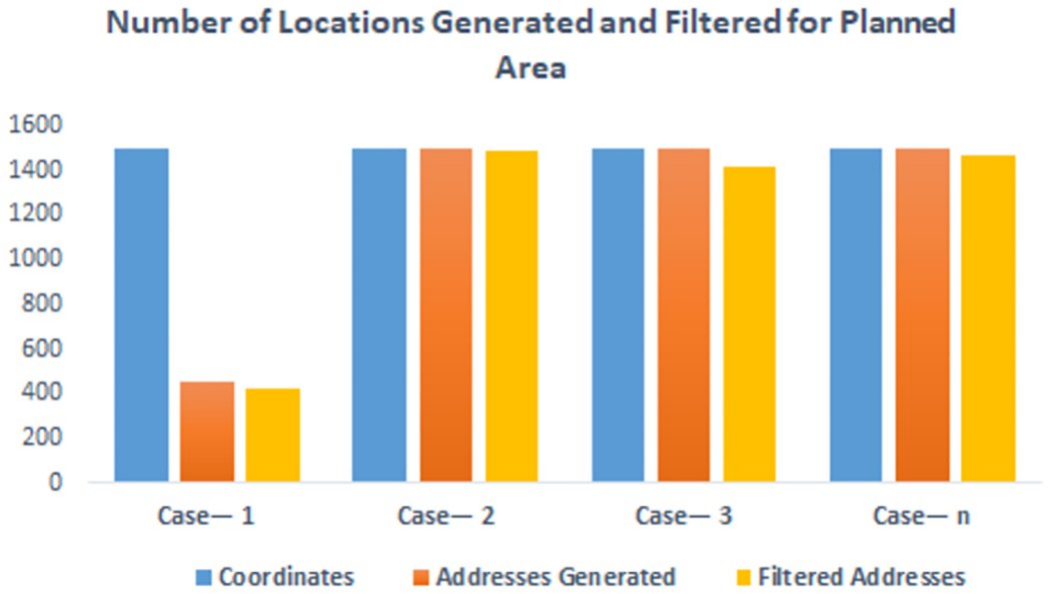
Result on planed area. Comparison of number of locations generated by location extraction module for planned Area.

**Fig 5:**
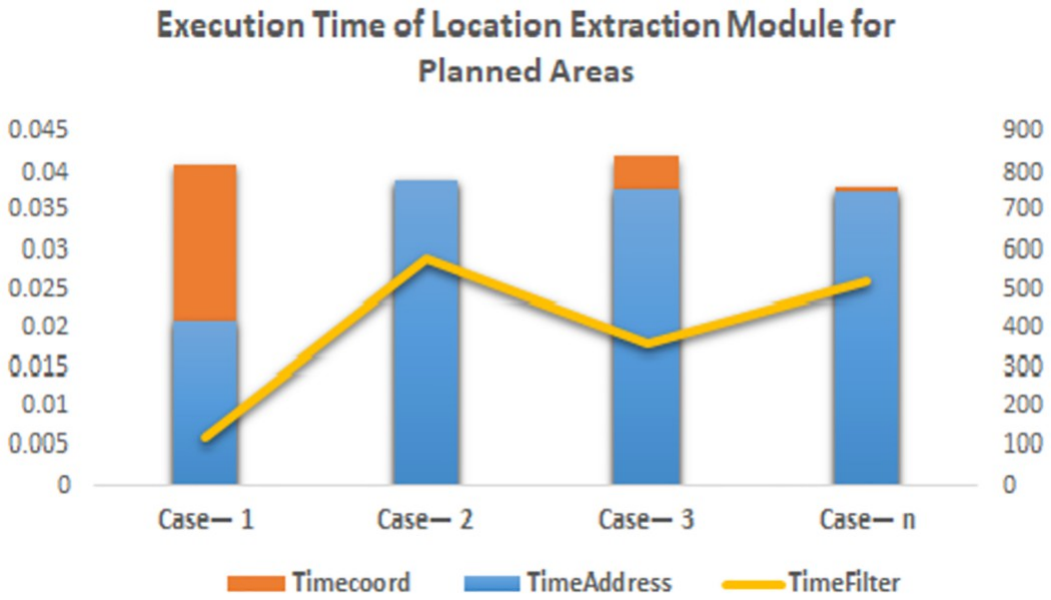
Execution time. Comparison of execution time of each module for planned area.

These results are depicted in Figs 4 and 5. Fig 4 represents the distribution of location extraction across all three component of location extraction module. Fig 5 depicts time taken by coordinates generation address generation and address filtration module. The execution time for address generation is plotted on the secondary axis and is represented as the most time-consuming activity. This is because address generation depends on reverse geocoding calls to the mapping service provider API.

### Experimentation procedure for unplanned areas

To depict unplanned areas, we opted for a city featuring various planned divisions that, geographically, vary in size. Some divisions are smaller, others larger, making it challenging to directly obtain minimum and maximum coordinates. Hence, we segmented these sectors into approximately rectangular or square shapes and extracted coordinates using a method akin to that used for planned regions. Table 5 represents, the number of coordinates, addresses, and filtered addresses generated along with time to generate them. It is clearly represented in table that results of unplanned area location extraction module are similar to that of planned area location extraction module. It strongly support the generalization aspect of SDECT.

**Fig 6:**
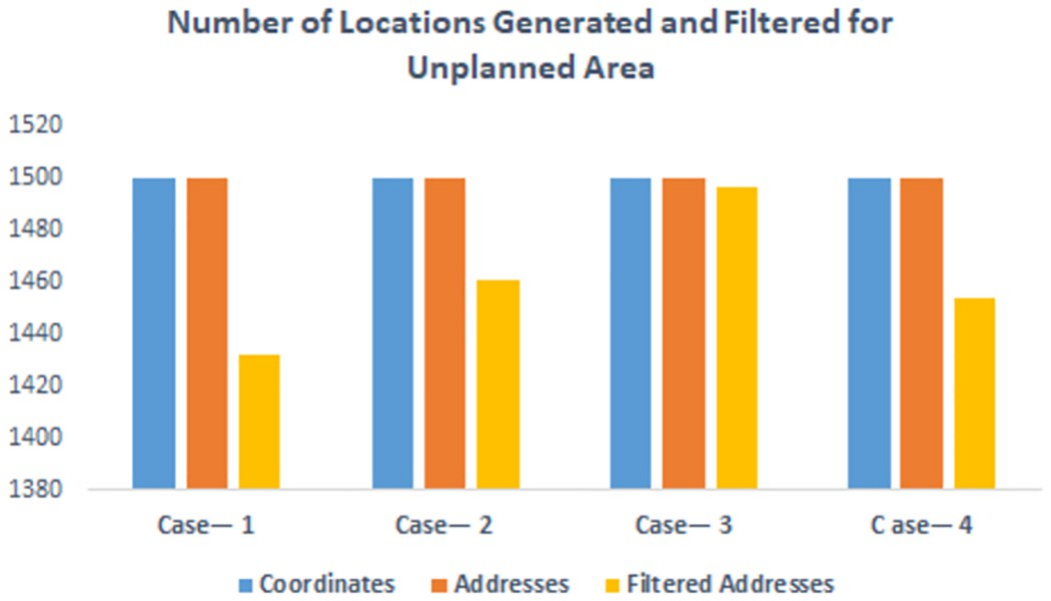
Result on unplanned area. Comparison of number of locations generated by location extraction module.

**Fig 7:**
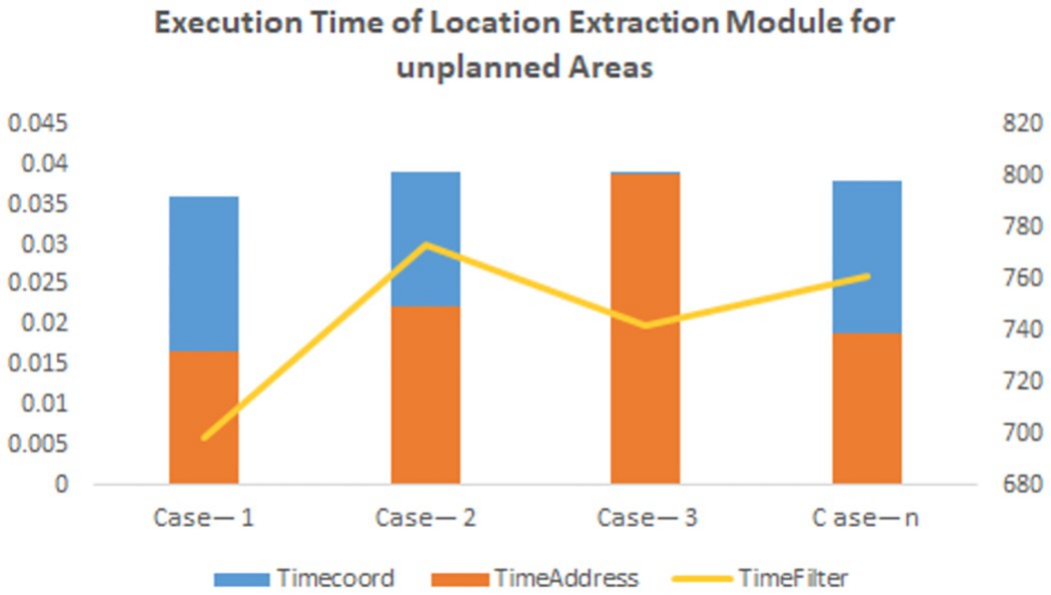
Execution time. Comparison of execution time of each module for unplanned area.

Fig 6 illustrates the distribution of location extraction across all three components of the location extraction module in unplanned areas, while Fig 7 showcases the time taken by the coordinates generation, address generation, and address filtration modules. The similarity of these results to those in planned areas further solidifies the applicability of SDECT in both scenarios.

### Experiments on graph generation module

The Table 6 displays the outcomes of experiments depicting graphs generated from delivery point locations extracted through the location extraction module. This module further derives a distance matrix for each graph, saved in distinct files. The data in Table 6 reveals the creation of eight graph sizes to showcase SDECT’s functionality. Notably, the size parameter is customizable to fit specific requirements. Each graph is labeled as VRP-n, denoting its extraction for the vehicle routing problem, with ‘n’ indicating the total number of locations within the graph. These graphs encompass a depot location, serving as the initial point, distinct from the remaining delivery locations (n-1). The time taken for generation varies based on graph size: smaller graphs, like VRP-20, take 0.07 seconds, whereas larger ones, such as VRP-350, require 22.97 seconds. This duration represents the generation time for a single graph. The ‘total record’ column indicates the generation of 100,000 graphs for each graph size. The choice of CSV file format aims to highlight the dataset’s maximum memory usage; using a lightweight file format can significantly reduce memory consumption. These experiments cover eight diverse graph sizes, implying potential variations in SDECT performance based on size and structure.

It is important to note that the experiments are conducted on eight different graph sizes. It is possible that the algorithm performance may vary depending on graph sizes and structures.

## Conclusion

In conclusion, our Spatial Data Extraction and Curation Tool (SDECT) offers a novel approach for generating real-world compatible data for DRL-based vehicle routing models. This tool addresses the critical need for high-quality datasets, enabling practical application of advanced techniques in route optimization that extends beyond vehicle routing, benefiting various industries reliant on real-world location data. For instance, telecommunications companies can optimize cell tower placement, design efficient fiber optic cable routes, plan drone-based internet delivery, and deploy emergency communication networks. Moreover, SDECT enhances maintenance operations by optimizing routes for repair crews, increasing efficiency throughout the telecommunications sector. However, it’s essential to acknowledge the challenges that SDECT faces during the dataset extraction phase. Acquiring accurate real-world data can be costly, and striking the right balance between security and user privacy for location information is crucial. Additionally, the dynamic nature of traffic and unforeseen events necessitates constant adaptation during data extraction. These challenges present opportunities for future improvement of the proposed tool.
